# The role of cytoreductive nephrectomy and systemic therapy in the management of tumour thrombus in patients with metastatic renal cell carcinoma

**DOI:** 10.1038/s41416-023-02166-5

**Published:** 2023-03-01

**Authors:** Abhenil Mittal, Esmail Al-Ezzi, Xuan Li, Brian Moloney, Brooke Wilson, Pavlina Spiliopoulou, Srikala Sridhar, Nazanin Fallah-Rad, Peter Chung, Robert James Hamilton, Martin O’malley, Aaron R. Hansen

**Affiliations:** 1 Division of Medical Oncology and Hematology, Princess Margaret Cancer Centre, University Health Network, Toronto, ON Canada; 2 Department of Biostatistics, Princess Margaret Cancer Centre, University Health Network, Toronto, ON Canada; 3 Division of Abdominal Radiology, Joint Department of Medical Imaging, University Health Network, Toronto, ON Canada; 4 Radiation Oncology Department, Princess Margaret Cancer Centre, University Health Network, Toronto, ON Canada; 5 Division of Urologic Oncology, Princess Margaret Cancer Centre, University Health Network, Toronto, ON Canada; 6 Division of Cancer Services, Princess Alexandra Hospital, Metro South Health, Brisbane, QLD Australia; 7 Department of Medicine, University of Toronto, Toronto, ON Canada; 8 Faculty of Medicine, University of Queensland, Brisbane, Australia

**Keywords:** Renal cell carcinoma, Renal cell carcinoma

## Abstract

**Background:**

Outcomes for patients with metastatic renal cell carcinoma (mRCC) and tumour thrombus remain poor. Recent data suggest limited role for cytoreductive nephrectomy (CN) and data on thrombus response to systemic therapy (ST) is scarce. Here, we describe response and survival of patients with de novo mRCC and thrombi treated with ST with or without CN.

**Methods:**

Demographics, disease characteristics and survival of patients with de novo mRCC were collected. Progression-free survival (PFS) and overall survival (OS) in months (m) was calculated using the Kaplan–Meier method (log-rank).

**Results:**

Between 2002 and 2019, 226 patients with mRCC were identified, 64 (28.3%) had tumour thrombus out of which 18 (28.1%) received only ST. Among 12 evaluable patients, thrombus response, stability and progression were seen in 3 (25%), 6 (50%) and 3 (25%) patients, respectively. Median OS was similar for patients with and without tumour thrombus treated with systemic therapy alone [OS: 12.1 m (8.8–27.7) vs. 13.9 m (7.9–21.5), *p* = 0.87]. CN predicted for better OS in patients with tumour thrombus [OS: 29.4 m (17.4–48.9) vs. 12.1 m (8.8–27.7), *p* = 0.01].

**Conclusion:**

In this retrospective series of patients with mRCC and tumour thrombus, addition of CN to ST improved outcomes. Validation of these findings with contemporary regimens is needed.

## Introduction

Venous invasion has been associated with poor prognosis in patients with localised RCC [1–3]. Extent of venous involvement has been recognized as an important prognostic factor in the eighth edition of the American Joint Committee on Cancer (AJCC) TNM system. Specifically, T3a, T3b, and T3c tumours invade the renal vein or its branches; the inferior vena cava (IVC) below the diaphragm; and the wall of the IVC or grossly extending into the IVC above the diaphragm, respectively [2, 4, 5].

Although the prognostic impact of venous invasion in localised RCC is well defined, the implications are uncertain in the metastatic setting. The established standard of care for patients with metastatic RCC (mRCC) presenting with venous thrombosis has been nephrectomy, based on data extrapolated from localised RCC or derived from older studies showing a survival benefit of cytoreductive nephrectomy (CN) in the metastatic setting [6, 7]. However, the role of surgery has been challenged by the CARMENA and SURTIME trials, which demonstrated no benefit for CN in the setting of metastatic disease [8, 9]. Moreover, some case series and case reports have described response of tumour thrombus to tyrosine kinase inhibitors (TKI) and immunotherapy (IO) in mRCC when used preoperatively in the curative setting [10–18]. Given the increasing number of effective systemic therapy options in mRCC and lack of a definite survival benefit of CN, there is a need to revisit the role of CN in patients with mRCC and tumour thrombus.

The primary objective of this single centre retrospective study was to describe the clinical characteristics, response to systemic therapy and outcomes of patients with mRCC and tumour thrombus. We also explored the impact of tumour thrombus on survival in the mRCC cohort who did not have CN. As a secondary objective, impact of CN was evaluated in a subgroup of patients with tumour thrombus who had this procedure. We hypothesised that effective systemic therapy may address the negative prognostic impact of tumour thrombus and CN might provide additional benefit to patients with tumour thrombus beyond systemic treatment.

## Methods

### Data extraction

Records of patients with de novo mRCC treated at Princess Margaret Cancer Center between January 2002 and January 2019 were reviewed retrospectively. Patients with both clear cell and non-clear cell RCC were eligible for inclusion. After review of baseline imaging, patients who had tumour thrombus and had either undergone CN or had been treated with systemic therapy alone were identified. This was our primary cohort of interest and we collected clinical information including age, sex, Karnofsky Performance Status (KPS), number and sites of metastasis at baseline, histological subtype of RCC, extent of venous thrombosis including Mayo category [19] and laboratory data including haemoglobin, neutrophils, platelets, calcium and lactate dehydrogenase (LDH) to assess International Metastatic RCC Database Consortium (IMDC) score. Also, data on type of systemic therapy (TKI, IO, chemotherapy, or their combinations) and response in both tumour and thrombus, anticoagulation as well as date of progression on first line systemic therapy and death were collected. Radiological assessment of response to systemic therapy in the primary tumour, thrombus and metastatic sites was reported in accordance with RECIST v1.1 criteria [20]. Additionally, a cohort of mRCC patients without tumour thrombus who received only systemic therapy was identified (as a comparator group) and clinical and laboratory data as defined above were collected. Mayo classification [19] was used to assess the impact of level of thrombus on survival in patients who had CN (mayo category 0/I/II vs. III/IV). Progression-free survival (PFS) on first line systemic therapy for mRCC was defined from date of start of systemic therapy to clinical/radiological progression or death whichever came earlier. Overall survival (OS) was defined from date of diagnosis to date of death. The study was approved by the Institutional Research Ethics Board (REB) of Princess Margaret Cancer Centre.

#### End points and comparisons

The primary end point of the study was investigator assessed best overall imaging response in tumour thrombus to systemic therapy and qualitative comparison with response at other metastatic sites. Secondary end points included evaluation of PFS (on 1^st^ line therapy) and OS in patients not undergoing CN (with and without thrombus) including impact of various clinical variables on outcomes apart from tumour thrombus. A co-secondary end point was estimation of OS in patients with tumour thrombus with and without CN.

#### Statistical analysis

Baseline demographics, clinical and laboratory characteristics were described using absolute numbers and percentages for categorical variables and median with inter-quartile range for continuous variables. Comparisons between baseline characteristics were made using Mann–Whitney *U*-test (considering non-normal data distribution) for continuous variables and Chi-squared test or Fisher exact test (as appropriate) for categorical variables. Survival analysis to determine PFS and OS was done using Kaplan–Meier (KM) method and the survival curves were statistically compared with each other using log-rank method. The effect of various baseline clinical parameters on PFS and OS was determined using univariate and multivariate cox regression models. Any variable with a *p*-value < 0.1 on univariate analysis was tested in a multivariate model. All analysis were done using R, version 3.6.1 (R Foundation for Statistical Computing, Vienna, Austria) and *p*-value of <= 0.05 was considered significant.

## Results

### Demographics and definition of study groups

**Fig. 1:**
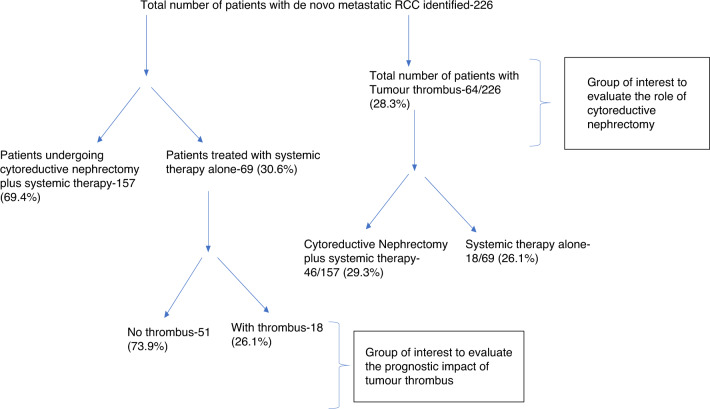
Distribution of patients in the cohort.

Among the 226 de novo mRCC patients treated at Princess Margaret Cancer Center during the study period, (Fig. 1), 157 (69.6%) underwent CN, and 69 (30.4%) received only systemic therapy.

Among all patients, 64 (28.3%) had a tumour thrombus; 46 (71.8%) patients with a tumour thrombus had undergone CN and 18 (28.2%) were treated with systemic therapy alone. Three patients (4.7%) had intracardiac extension of thrombus. Baseline characteristics of patients with tumour thrombus are described in Table 1. Median age of patients with tumour thrombus was 58.6 years (IQR 50–66.4), majority were males (*N* = 49, 77%) and had clear cell RCC (*N* = 44, 69%) (Table 1). Most of the patients were classified as intermediate or poor risk by IMDC risk stratification (*N* = 61, 95%). Three fourth (*N* = 48, 75%) of patients had more than one site of metastatic disease at presentation with median number of sites being two (range 1–4). Most common site of metastatic disease was lung (*N* = 51/64, 79.7%) followed by non-regional lymph nodes (*N* = 39/64, 60.9%), bone (*N* = 24/64, 37.5%) and liver (*N* = 17/64, 26.6%). Brain metastases were seen in four patients at baseline (6.2%) (Table 1). Forty patients (62.5%) had a KPS of >= 80%. A total of 30 patients (46.9%) received anticoagulation at the discretion of the treating physician.

**Table 1:** Baseline characteristics in all patients with venous thrombosis.

Covariate *N* (%)	Full sample (*n* = 64)
Age (years)	
Median (Q1, Q3)	58.6 (50.0, 66.4)
Gender	
Female	15 (23)
Male	49 (77)
Histology	
Clear cell	44 (69)
Non-clear cell	19 (30)
Unclassified tumour	1 (2)
Number of metastatic sites	
One	16 (25)
Two	23 (35.9)
Three	17 (26.6)
Four	8 (12.5)
Location of metastasis	
Non-regional lymph nodes	39 (60.9)
Lung	51 (79.7)
Brain	4 (6.2)
Liver	17 (26.6)
Bone	24 (37.5)
KPS	
<80%	24 (37.5)
>=80%	40 (62.5)
IMDC category	
Favourable	3 (5)
Intermediate/poor	61 (95)
Mayo classification	
0	31 (50)
I	7 (11)
II	9 (15)
III	11 (18)
IV	4 (6)
Systemic therapy	
Sunitinib	48 (75)
Sorafenib	5 (7.8)
Pazopanib	2 (3.1)
Chemotherapy	3 (4.6)
Nivolumab-ipilimumab	5 (7.8)
Bevacizumab Erlotinib	1 (1.6)

*KPS* Karnofsky Performance Score, *IMDC* International Metastatic Renal cell Carcinoma Database Consortium.

#### Response and outcomes of patients with tumour thrombus treated with systemic therapy

Seventeen out of 18 (94.4%) patients with tumour thrombus received treatment with a anti vascular endothelial growth factor tyrosine kinase inhibitor (VEGF TKI) sunitinib *n* = 14, pazopanib *n* = 2, sorafenib *n* = 1) and one patient with medullary histology received chemotherapy with gemcitabine and cisplatin. Twelve out of 18 patients were eligible for response assessment in the thrombus. Three (25%) had a partial response (PR), six (50%) had stable disease (SD) and three (25%) had evidence of progression of thrombus after systemic therapy. For the remaining six patients, response assessment could not be done due to segmental thrombi in two patients, non-availability of response assessment scan in the electronic system in two patients, non-contrast scan in one patient and baseline MRI, which could not be compared with a response assessment CT scan in one patient. In terms of response at other sites, five of 18 patients had a partial response (27.7%), 8 (44.4%) had stable disease and 5 (27.7%) had disease progression as best response. Among nine patients who had evidence of response/stabilisation in the thrombus, eight patients (88.8%) had evidence of response/stable disease at other sites as well.

#### Evaluation of prognostic impact of tumour thrombus in mRCC patients treated with systemic therapy alone

**Fig. 2:**
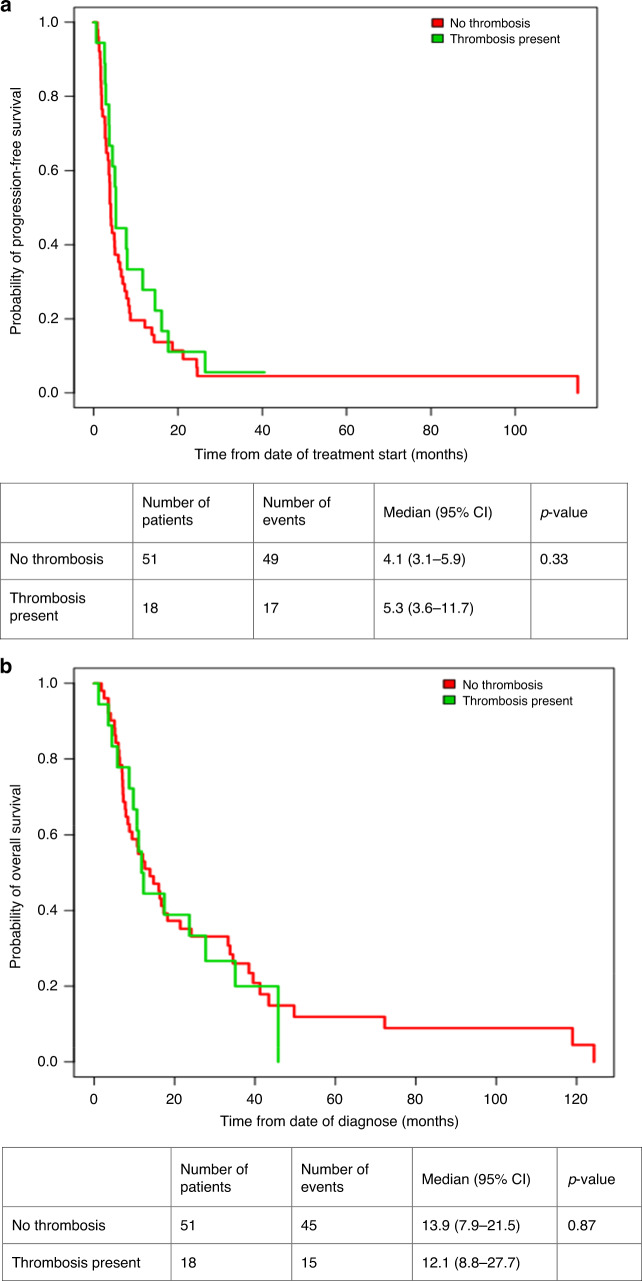
Survival outcomes in patients with and without thrombosis. **a** Progression-free survival (thrombosis vs. no thrombosis). **b** Overall survival (thrombosis vs. no thrombosis).

Baseline clinical characteristics among patients with and without thrombosis who received only systemic therapy are shown in Table 2. There were no statistically significant differences between both groups. Survival outcomes for patients who received only systemic therapy (*N* = 69) were similar irrespective of presence of tumour thrombus. Median PFS was 5.3 months for patients with thrombosis (95% CI 3.6–11.7) vs. 4.1 months for patients without thrombosis (95% CI 3.1–5.9), *p* = 0.33 (Fig. 2a). Median OS was 12.1 months (95% CI: 8.8–27.7) for patients with thrombosis and 13.9 months (95% CI: 7.9–21.5) for patients without thrombosis, *p* = 0.87 (Fig. 2b).

**Table 2:** Baseline characteristics between patients with thrombosis and no thrombosis treated with systemic therapy only.

Covariate	Full sample (*n* = 69)	No thrombosis (*n* = 51)	Thrombosis present (*n* = 18)	*p*-value^a^
Age				0.71
Median (Q1, Q3)	62.4 (55.9,71.1)	63.3 (56.0,73.2)	61.4 (55.1,69.6)	
Gender				0.53
Female (*n*, %)	17 (25)	14 (27)	3 (17)	
Male (*n*, %)	52 (75)	37 (73)	15 (83)	
Histology				0.64
Clear cell (*n*, %)	48 (70)	34 (67)	14 (78)	
Non-clear cell (*n*, %)	12 (17)	9 (18)	3 (17)	
Unclassified (*n*, %)	9 (13)	8 (16)	1 (6)	
IMDC category				1
Intermediate (*n*, %)	35 (51)	26 (51)	9 (50)	
Poor (*n*, %)	34 (49)	25 (49)	9 (50)	
No of metastatic sites				0.36
1 (*n*, %)	17 (24.7)	14 (27.4)	3 (16.7)	
>1 (*n*, %)	52 (75.7)	37 (72.6)	15 (83.3)	
KPS				0.66
<80% (*n*, %)	22 (31.9)	17 (33.3)	5 (27.7)	
>=80% (*n,*%)	47 (68.1)	34 (66.7)	13 (72.3)	
Systemic therapy				
Sunitinib (*n*, %)	47 (68.1)	33 (64.7)	14 (77.8)	
Sorafenib (*n*, %)	3 (4.3)	2 (3.9)	1 (5.5)	
Pazopanib (*n*, %)	4 (5.8)	2 (3.9)	2 (11.1)	
Chemotherapy (*n*, %)	4 (5.8)	3 (5.9)	1 (5.5)	
Nivolumab-ipilimumab (*n*, %)	11 (15.9)	11 (21.6)	0	

*IMDC* International Metastatic Renal Cell carcinoma Database Consortium, *KPS* Karnosfsky Performance Score.

^a^The *p*-value for comparison between No thrombosis and thrombosis group.

Non-clear cell histology (HR 2.25, 95% CI 1.17–4.33, *p* = 0.015), IMDC poor risk category (HR 1.64, 95% CI 1.0–2.68), *p* = 0.049) and KPS < 80% (HR 1.77, 95% CI 1.04–2.99, *p* = 0.041) predicted for inferior PFS on univariate analysis. Among these, only non-clear cell histology was an independent predictor on multivariate analysis (HR 2.53, 95% CI 1.3–4.9, *p* = 0.006). Similarly for OS, non-clear cell histology (HR 1.97, 95% CI 1.02–3.8, *p* = 0.04), KPS < 80% (HR 1.65, 95% CI 0.95–2.87, *p* = 0.09) and IMDC poor risk category (HR 2.32, 95% CI 1.37–3.93, *p* = 0.002) predicted for inferior OS on univariate analysis. Among these, IMDC poor risk category (HR 2.47, 95% CI 1.35–4.49, *p* = 0.003) and non-clear cell histology (HR 2.46, 95% CI 1.25–4.84, *p* = 0.009) emerged as independent predictors of inferior OS on multivariate analysis. Tumour thrombus did not have an independent impact on either PFS (HR 0.76 95% CI 0.44–1.33, *p* = 0.34) or OS (HR 1.05, 95% CI 0.58–1.90, *p* = 0.87).

#### Evaluation of impact of CN on outcomes of patients with tumour thrombus

Clinical characteristics between patients with tumour thrombus who did or did not undergo CN were similar (Table 3). All patients received systemic therapy with an anti-VEGF TKI [*N* = 39 (84.78%], combination immunotherapy with nivolumab plus ipilimumab [*N* = 5, (8.69%)] or chemotherapy [*N* = 2, (4.34%)] (Table 3).

**Table 3:** Baseline characteristics between patients with thrombosis who underwent cytoreductive nephrectomy (CN) and did not undergo CN.

Covariate	Full sample (*n* = 64)	No surgery (*n* = 18)	Cytoreductive nephrectomy (*n* = 46)	*p*-value^a^
Age				0.087
Median (Q1, Q3)	58.6 (50.0,66.4)	61.4 (55.1,69.6)	57.1 (48.6,63.8)	
Gender				0.53
Female (*n*, %)	15 (23)	3 (17)	12 (26)	
Male (*n*, %)	49 (77)	15 (83)	34 (74)	
Histology				0.11
Clear cell (*n*, %)	44 (69)	14 (78)	30 (65)	
Non-clear cell (*n*, %)	19 (30)	3 (17)	16 (35)	
Unclassified tumour (*n*, %)	1 (2)	1 (6)	0 (0)	
No of metastatic sites				
>1 (*n*, %)	48 (75)	15 (83.3)	33 (68.8)	0.52
1 (*n*, %)	16 (25)	3 (16.7)	13 (27.1)	
KPS				0.61
<80 (*n*, %)	24 (37.5)	5 (27.8)	10 (21.7)	
>=80 (*n*, %)	40 (62.5)	13 (72.2)	36 (78.3)	
IMDC category				0.55
Favourable (*n*, %)	3 (5)	0 (0)	3 (7)	
Intermediate/poor (*n*, %)	61 (95)	18 (100)	43 (93)	
Mayo classification				0.4
0 (*n*, %)	31 (50)	13 (72.2)	20 (43)	
I (*n*, %)	7 (11)	0 (0)	7 (15)	
II (*n*, %)	9 (15)	2 (12)	7 (15)	
III (*n*, %)	11 (18)	2 (12)	9 (20)	
IV (*n*, %)	4 (6)	1 (6)	3 (7)	
Systemic therapy				
Sunitinib (*n*, %)	48	14	34	
Sorafenib (*n*, %)	5	1	4	
Pazopanib (*n*, %)	2	2	0	
Chemotherapy (*n*, %)	3	1	2	
Nivolumab-ipilimumab (*n*, %)	5	0	5	
Bevacizumab Erlotinib (*n*, %)	1	0	1	

*IMDC* International Metastatic Renal Cell carcinoma Database Consortium, *KPS* Karnofsky Performance Score.

^a^Comparison between surgery and no surgery group.

**Fig. 3:**
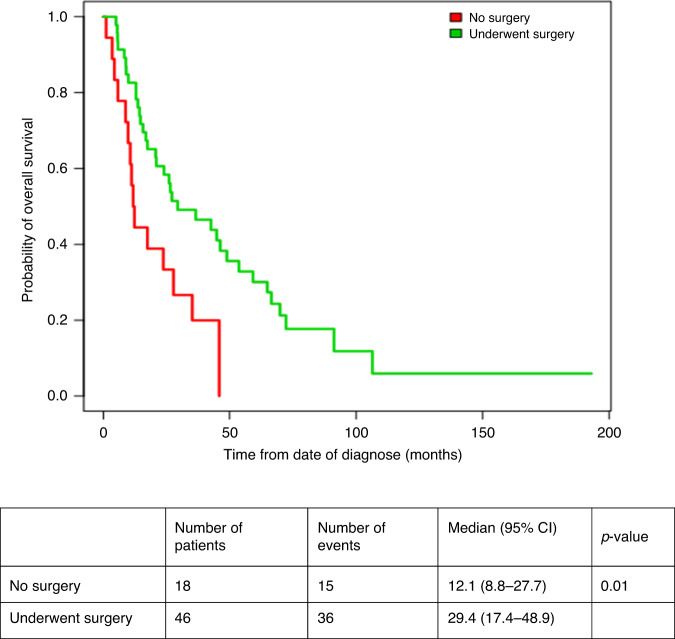
Overall survival for patients with thrombosis with and without CN.

Median OS was significantly better in patients who underwent CN (29.4 months (95% CI: 17.4–48.9) vs. 12.1 months (95% CI: 8.8–27.7, *p* = 0.01, Fig. 3) for systemic therapy only. Level of thrombus in the venous system (below or above hepatic veins, i.e., Mayo classification 0,I,II vs. III,IV) did not affect OS [24 months (95% CI 14.07–34) for level 0/I/II and 36.5 months (95% CI 12.8–60.3) for level III/IV, *p* = 0.23].

On Cox regression analysis, CN (HR 0.43 95% CI 0.23–0.82, *p* = 0.011) and KPS > 80% (HR 0.49, 95% CI 0.26–0.93, *p* = 0.03) predicted for better OS, whereas there was no significant association with number of metastatic sites (HR 1.3, 95% CI 0.96–1.76, *p* = 0.11). On multivariate analysis, both CN (HR 0.42, 95% CI 0.22–0.8, *p* = 0.009) and KPS > 80% (HR-0.46, 95% CI 0.24–0.89, *p* = 0.89) were independent predictors of better OS.

## Discussion

RCC is a biologically unique tumour with a tendency for vascular invasion, which at presentation can be seen in around 5–10% of patients with localised RCC;[21] and 29–56% of mRCC [22, 23]. In our series, we found a similarly high percentage of mRCC patients having tumour thrombus at diagnosis (28.3%). Only three patients (4.3%) had intracardiac extension of thrombus, which is consistent with previously reported literature [24, 25].

The prognostic impact of tumour thrombus in the presence of metastatic disease in patients treated with targeted therapy without surgery remains unknown. With the publication of CARMENA and SURTIME trials [8, 9], where CN did not demonstrate a survival benefit in the setting of TKI therapy, surgery is no longer considered standard of care. In this single center retrospective study done at a tertiary referral center in Canada, we found that the key prognostic factors were histology (non-clear cell having inferior outcomes) and IMDC category but tumour thrombus itself does not have a prognostic impact in mRCC. Notwithstanding, among patients with tumour thrombus, CN improved survival.

To our knowledge, this is the largest cohort study to report PFS and OS outcomes and response rate in patients with tumour thrombus receiving TKI. Our data suggest that the response in tumour thrombus to TKI therapy is similar to overall tumour response observed in randomised phase III trials in the metastatic setting and correlates well with response at other sites [26, 27]. Previous studies have looked at using anti-VEGF TKI’s in the pre-surgical setting in non-metastatic RCC to shrink tumour thrombus and facilitate surgery, with variable results. A previous study by Field et al. reported on 53 patients (19 received preoperative Sunitinib and 34 upfront surgery). In each group, nine patients had mRCC [15]. Overall, partial response in thrombus was seen in 27.8% of patients, similar to the present study. Although they demonstrated an improvement in cancer-specific survival (CSS) with sunitinib, this benefit was restricted to patients with metastatic disease demonstrating that sunitinib may be an effective treatment option in patients with mRCC with tumour thrombus. Another study by Cai et al. did not show significant improvement in survival with neoadjuvant sunitinib or sorafenib in non-metastatic patients with only one patient having a reduction in tumour thrombus [16]. Okamura et al. and Bigot et al. reported higher responses in tumour thrombus (43–44%) in the non-metastatic setting with response in primary tumour mirroring response in the thrombus but survival was not reported [17, 18].

We found that the presence of thrombus did not negatively impact survival in patients who were treated with systemic therapy alone. A study by Goetzl et al. also did not find any prognostic relevance of tumour thrombus in patients with metastatic RCC, however, all patients in this study underwent CN and details about systemic therapy were not available [23]. Since this study was published in 2004, it is unlikely that any of these patients would have received an anti-VEGF TKI or immunotherapy. Since 2004, none of the published studies have reported on the prognostic relevance of tumour thrombus in patients with mRCC. The aforementioned study by Field et al. showed that treatment with neoadjuvant sunitinib could improve outcomes in patients with mRCC with tumour thrombus [15]. Although we could not isolate the impact of anti-VEGF TKI in our cohort as all patients received systemic therapy, the similar prognosis of patients with and without tumour thrombus could be attributed to effective systemic therapy or because tumour thrombus is not prognostic for outcomes.

We observed that patients with non-clear cell histology had inferior survival. Patients with non-clear cell RCC (particularly with sarcomatoid and rhabdoid features) have been shown to have durable response to dual checkpoint blockade but tend to respond poorly to anti-VEGF TKI [28]. Majority of the patients in our cohort received an anti-VEGF TKI as their primary systemic therapy and importance of non-clear cell histology as an independent predictor of outcome needs validation in larger contemporary cohort of patients treated with checkpoint inhibitors.

IMDC risk category remains relevant in daily practice and is widely used in contemporary clinical trials as a risk stratification tool. Patients classified as poor risk by IMDC had shorter OS in our study, thus highlighting its importance for risk stratification. Although newer models have been proposed, they are yet to be validated [29] and IMDC remains the most widely accepted standard for clinical risk stratification in mRCC. The slightly inferior survival outcomes for patients treated with anti-VEGF TKI in our study compared to the seminal paper by Heng et al. [30] could be explained by higher proportion of patients with poor risk disease in our cohort. Taken together, these findings suggest that classical prognostic factors like IMDC risk and histology are more relevant than tumour thrombus in determining outcomes in these patients and should be considered when planning management.

We also investigated the role of CN in patients with tumour thrombus and found that it significantly improved OS. Although better performance status was associated with improved outcomes, impact of CN was seen independent of KPS and baseline characteristics were balanced between the two groups with no statistically significant differences. Therefore, an actual benefit of CN in this high-risk group is possible but needs confirmation in a larger cohort of patients. Historically, CN was the historical standard of care for patients with mRCC treated in the interferon era based on a survival benefit in randomised trials [6, 7], however, the publication of CARMENA and SURTIME trials [8, 9] challenged this paradigm. Despite these trials, real world data continue to show benefit from CN in patients treated with VEGF TKI’s and immunotherapy [31–33] and it has remained a standard of care in patients with venous thrombus. Lenis et al. queried the National Cancer Database (NCDB) and found that patients with renal vein or infradiaphragmatic IVC thrombus had better outcomes with CN compared to patients with supradiaphragmatic thrombus where there was no impact [34]. Similar findings were reported by Abel et al. where they found supradiaphragmatic tumour thrombus correlated with inferior survival and with early mortality [35]. However, the correlation between the extent of venous thrombosis and survival has not been demonstrated in other studies [36, 37]. The absence of correlation between extent of thrombosis and survival in our study could be due to very small number of patients with supradiaphragmatic thrombus extension (*N* = 3) and needs to be interpreted cautiously.

Although CN showed an OS benefit in our study, the optimal timing of CN remains a matter of debate. In a recent study from the IMDC, Bhindi et al. showed that patients who had deferred CN after sunitinib had better OS compared to sunitinib alone or upfront CN [38]. Bruijn et al. also found that deferred CN may be better compared to upfront CN in patients treated with sunitinib [39]. Even in patients with tumour thrombus, whether a strategy of delayed CN would be as effective as upfront CN remains unknown. None of these studies reported a separate subgroup of patients with venous thrombus. With consistent benefits seen with CN in patients with tumour thrombus in various retrospective studies including the current study, the optimal timing of surgery in this subset of patients is a pertinent question for future studies. With 72.7% (27.7% PR and 44.4% SD) patients achieving at least disease stabilisation with systemic therapy, deferred CN may be attractive and may allow for less extensive surgery after a period of systemic therapy.

Our study has several limitations. First, none of the patients with tumour thrombus who were treated with systemic therapy alone received either immunotherapy (IO) or a combination of IO and anti-VEGF TKI. The modern management of mRCC has moved towards combination IO (nivolumab-ipilimumab especially for IMDC intermediate and poor risk disease) [40] or IO plus anti-VEGF TKI’s (pembrolizumab/axitinib, nivolumab/cabozantanib and pembrolizumab/envatinib) [41–43] based on large randomised trials. Response rates ranging from 60–70% have been reported in these studies; however, none of these studies provide subgroup analyses for patients with tumour thrombus at baseline. Only a few case reports have described response in tumour thrombus when immunotherapy was used in the neoadjuvant setting [10–13] and response rates in tumour thrombus from combination IO or IO/anti-VEGF TKI as compared to anti-VEGF TKI alone are unknown, and should be studied in larger observational studies and/or randomised trials. Second, this is a single center retrospective cohort study from a large referral center, which could result in selection bias. Third, the small numbers of patients in many of the subgroups used for comparison has limited the power of the study to detect significant differences for many variables of interest. Additionally, the exclusion of 1/3rd (6/18) of the patients in the thrombus group receiving anti-VEGF TKI from evaluation of thrombus responses due to various reasons is also a potential confounder.

To conclude, in this large series of patients with mRCC with tumour thrombus treated with only systemic therapy, around one quarter of patients had evidence of response in thrombus. Similar survival between patients with and without tumour thrombus when treated with an anti-VEGF TKI suggests that such therapy is effective regardless of the presence of thrombus. Improvement in outcomes with CN suggests that nephrectomy should be carefully considered, and patients should be properly selected regardless of level of tumour thrombus. Larger studies with IO-IO and IO-VEGF-based combinations are required to understand the prognostic role of tumour thrombus in these patients and whether CN will play as important a role in patients who receive these more effective treatments.

## Data Availability

Spreadsheet containing deidentified data can be made available upon reasonable request from the corresponding author.
